# Interacting effects of insect and ungulate herbivory on Scots pine growth

**DOI:** 10.1038/s41598-020-79346-3

**Published:** 2020-12-18

**Authors:** Michelle Nordkvist, Maartje J. Klapwijk, La rs Edenius, Christer Björkman

**Affiliations:** 1 Department of Ecology, Swedish University of Agricultural Sciences, Uppsala, Sweden; 2 Department of Wildlife, Fish, and Environmental Studies, Swedish University of Agricultural Sciences, Umeå, Sweden

**Keywords:** Ecology, Ecological networks, Forest ecology

## Abstract

Most plants are subjected to damage from multiple species of herbivores, and the combined impact on plant growth can be non-additive. Since plant response to herbivores tends to be species specific, and change with repeated damage, the outcome likely depend on the sequence and number of attacks. There is a high likelihood of non-additive effects on plant growth by damage from mammals and insects, as mammalian herbivory can alter insect herbivore damage levels, yet few studies have explored this. We report the growth response of young Scots pine trees to sequential mammal and insect herbivory, varying the sequence and number of damage events, using an ungulate-pine-sawfly system. Combined sawfly and ungulate herbivory had both additive and non-additive effects on pine growth—the growth response depended on the combination of ungulate browsing and sawfly defoliation (significant interaction effect). Repeated sawfly herbivory reduced growth (compared to single defoliation) on un-browsed trees. However, on browsed trees, depending on when sawfly defoliation was combined with browsing, trees exposed to repeated sawfly herbivory had both higher, lower and the same growth as trees exposed to a single defoliation event. We conclude that the sequence of attacks by multiple herbivores determine plant growth response.

## Introduction

The growth of plants can be altered by herbivory, and the growth response depends, for example, on herbivore identity^1^, timing, and intensity of herbivore attack^2–5^. Plants are rarely subjected to herbivory by only a single species of herbivore. Induced responses initiated by one herbivore, can affect how plants respond to subsequent herbivory, with potential consequences for plant growth. Herbivore induced responses, e.g. changes in morphological or chemical traits of the plant, can affect the performance of a subsequent species of herbivore (trait-mediated effect), and thus the level of damage^6,7^ and eventually plant growth. An understanding of interactive effects of multi-species herbivory is of importance in e.g. forestry where multiple pest species are common, and trees are exposed to herbivory over longer periods of time.

Induced plant responses are often specific to the herbivore attacker^8,9^, which leads to the expectation that the sequence of attack by multiple herbivore species could alter the growth response^9,10^. Repeated herbivory is also an important factor affecting plant growth^5^, which implies that the number of attacks in a multi herbivore species scenario may be of importance. Hence, the outcome of herbivory by multiple species could depend on how herbivory is combined over time, yet there appears to be a lack of studies that actively manipulate specific combinations of herbivory to test the importance of the sequence and number of herbivory events for plant growth. Moreover, the effects of multi-species herbivory on plant growth has mainly been studied with a focus on the effect of taxonomically similar species (mainly insects^11,12^). Research on combined effects of herbivory by taxonomically different species, such as mammals and insects, have rarely been explored (although see Strauss, 1991, Muiruri et al.^7,13^). Since mammal–insect interactions often are highly asymetrical^14^, sequential^15^ and may induce different types of plant responses the outcome for plant growth is likely to differ from multi-insect attacks.

The effect of herbivory by multiple species on plant growth can be either additive, i.e. equalling the sum of the single herbivore effects, or non-additive, i.e. being larger (synergistic effect) or smaller (antagonistic effect) than the sum of the single herbivore effects. Since mammalian herbivory has been shown to both increase and decrease subsequent insect damage levels^4,6,7^, there is a possibility for non-additive effects on plant growth by combined attack by these types of herbivores. In a meta-analysis of 51 studies on effects of herbivory by multiple insect species, Stephens et al.^11^ showed that approximately three quarters of the interactions had additive effects on plant growth/biomass and the remaining 25% of the effects were antagonistic (i.e. less than additive). Temporally separated herbivores tended to cause dominantly additive interactions^11^, possibly due to indirect effects not being strong enough to cause non-independent impact on the plant. Temporal separation is common in mammal–insect interactions. In one of the few studies involving mammals and insects, Strauss^13^ showed that deer (*Odocoileus virginianus*) and weevil (*Blepharida rhois*) damage had antagonistic effects on Smooth sumac (*Rhus glabra*) re-growth. Antagonistic effects are likely when there is a negative indirect interaction between the herbivores, which is often the case in mammal–insect indirect interactions^15^. Antagonistic effects could also be due to priming (defences initiated by the first herbivore, rendering the plant prepared for coming attacks)^16,17^.

The aim of the present study was to explore the effect of sequential herbivory by taxonomically distant herbivores, i.e. insects and mammals, and to test whether the specific combination matters for plant growth, by varying the sequence and number of herbivory events (i.e. the frequency/intensity). To explore these aspects, we used Scots pine (*Pinus sylvestris*), the European pine sawfly (*Neodiprion sertifer*) and simulated ungulate browsing (i.e. clipping) as a study system. Browsing by ungulates and herbivory by sawflies commonly occur on the same pine trees but during different times of the year.

In order to study the effect of combined ungulate and sawfly herbivory on pine growth, we quantified the effect on height and radial growth of single and sequential sawfly and simulated ungulate herbivory and varied the sequence and number of herbivory events by the two herbivores. We formulated the following research questions:

- Q1. Is height growth and radial growth of pine trees affected by browsing and sawfly defoliation?
- Q2. Is pine height growth and radial growth affected by the sequence of browsing and sawfly defoliation?
- Q3. Is the combined effect of browsing and sawfly defoliation on pine height and radial growth additive, synergistic or antagonistic?

Both ungulate winter-browsing and pine sawfly defoliation has been shown to reduce growth of Scots pine^5,18–20^. Both intensity and timing of browsing can alter growth in Scots pine, and higher number of browsing events (i.e. more herbivory over several years) result in larger growth reduction^5^. Similarly, intensity of sawfly defoliation is an important determinant of the effect on pine growth: high intensity defoliation impedes growth more than low intensity defoliation^18^. Based on these results we expect both ungulate browsing and insect defoliation to lower both radial and height growth, and that higher number of events (i.e. more damage) would exacerbate that effect. In combination, we expect the outcome of ungulate browsing and sawfly herbivory to have either additive or antagonistic effects on pine growth, due to the temporal separation^11^ and priming^17^. However, as mammalian herbivory can have both negative and positive effects on insect performance and damage^15,21^, there is a potential for both antagonistic and synergistic effects.

## Results

### **Q1**

Is height growth and radial growth of pine trees affected by browsing and sawfly defoliation?

Mean (± standard deviation) starting height and diameter at 0.2 m and 0.75 m was 172.0 ± 23.6 cm, 42.0 ± 8.2 mm and 29.5 ± 7.8 mm, respectively.

### Browsing

**Figure 1:**
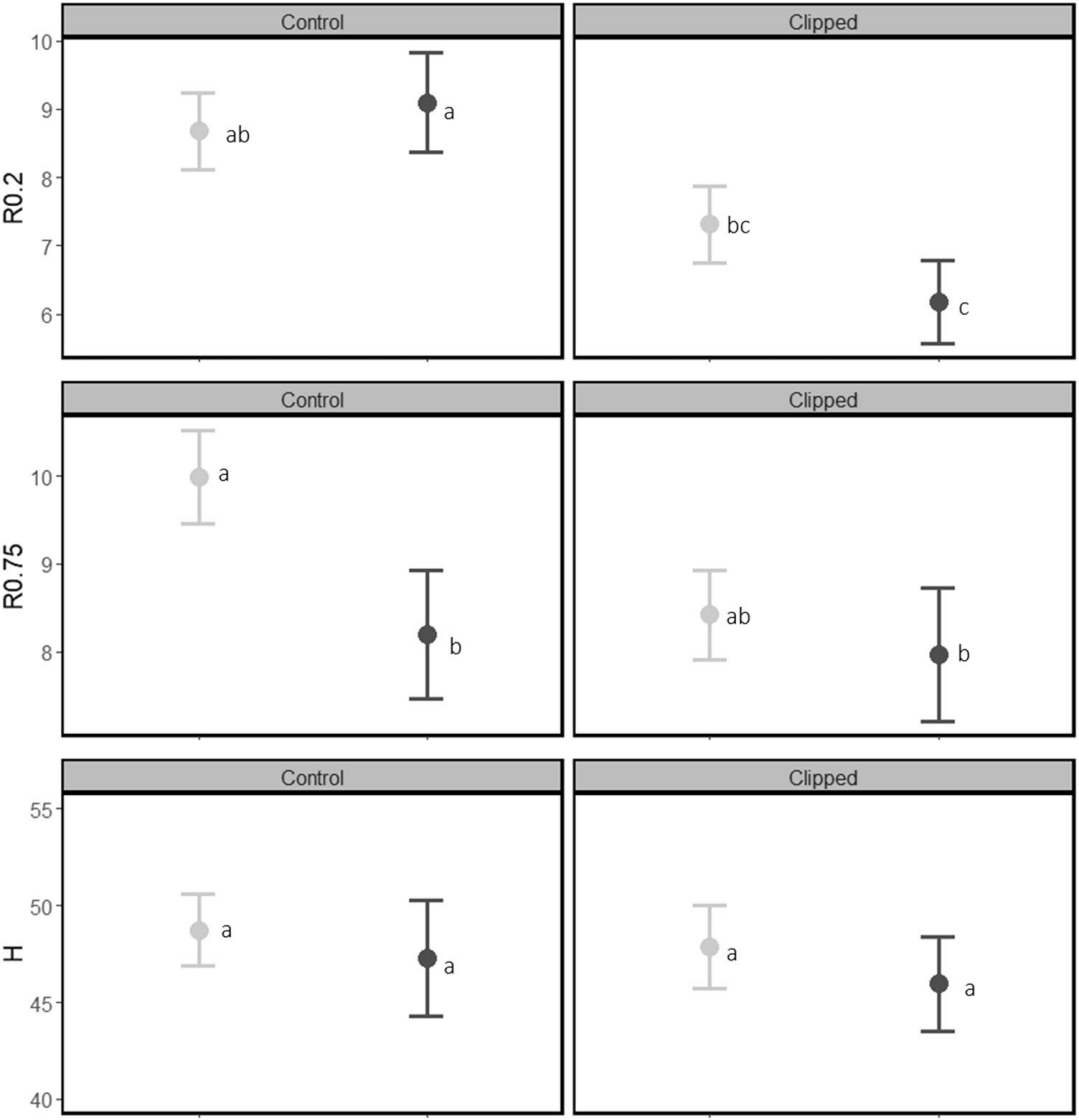
Total growth increment of pine trees in 2016 (mean ± SEM) at 0.2 m stem height (R0.2), 0.75 m stem height (R0.75) and in height (H). Vertical panels represent the browsing treatments: control (un-clipped) and clipped. Colours represent the sawfly treatments: no sawflies (light grey markers and error bars) or sawflies (dark green markers and error bars). Letters denote significant differences between individual treatments. Mean growth (left to right, top to bottom): 8.68, 9.10, 7.32, 6.18, 9.99, 8.19, 8.42, 7.96 mm and, 48.7, 47.3, 47.8, 46.0 cm.

**Figure 2:**
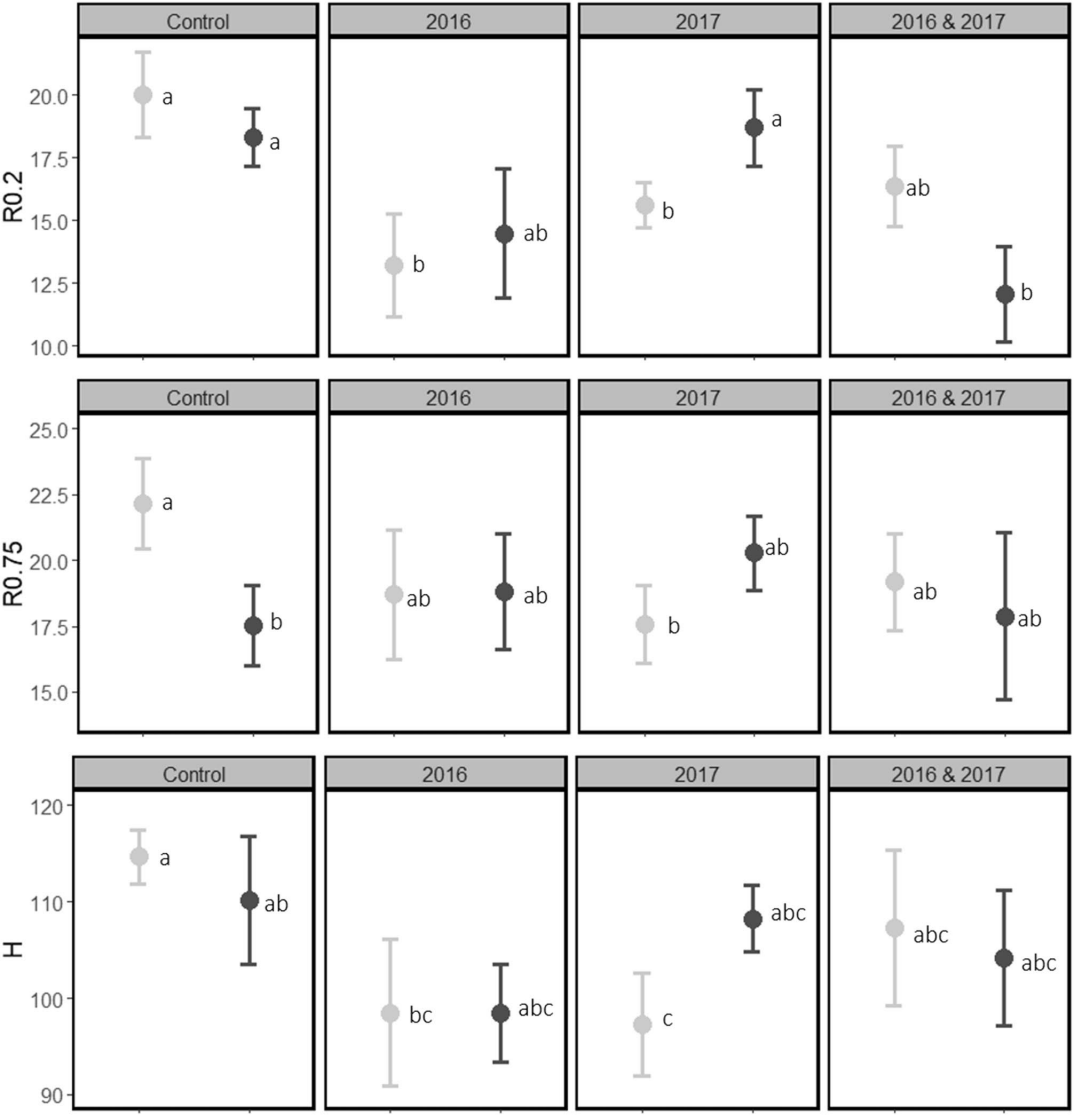
Total growth increment of pines trees in 2017 (mean ± SEM) at 0.2 m stem height (R0.2), 0.75 m stem height (R0.75) and in height (H). Vertical panels represent the browsing treatments: control (un-clipped), clipped 2016, clipped 2017 and clipped 2016 and 2017. Colours represent the sawfly treatments: sawflies once (light grey markers and error bars) or sawflies twice (dark grey markers and error bars). Letters denote significant differences between individual treatments. Mean growth (left to right, top to bottom): 20.02, 18.31, 13.21, 14.47, 15.63, 18.69, 13.36 and 12.06, 22.15, 17.51, 18.69, 18.83, 17.57, 20.27, 19.17, 17.88 mm and, 114.7, 110.1, 98.5, 98.4, 97.3, 108.2, 107.3, 104.1 cm.

After the first year of the experiment clipping resulted in 2.1 mm lower radial growth at 0.2 m (χ^2^ = 13.3, *p* < 0.001), corresponding to a decrease of 23%, but had no effect on radial growth at stem height 0.75 m or on height growth (Table 1, Fig. 1), compared to un-clipped control trees. During the second year, clipping resulted in 22% lower radial growth at 0.2 m stem height (χ^2^ = 27.5, *p* < 0.001; Table 2; Fig. 2), 9% lower height growth (χ^2^ = 20.9, *p* < 0.001; Table 2; Fig. 2) but no effect on radial growth at 0.75 m (Table 2; Fig. 2), compared to un-clipped trees.

**Table 1:** ANOVA (type II) test results for linear mixed model testing the growth response (height and diameter (at both 0.2 m and 0.75 m stem height)) in relation to starting height/diameter and the herbivory treatments (browsing and insects) and their interaction, year one (2016) of the experiment. Significant p-values are marked in bold.

Herbivory	Diameter growth 0.2				Diameter growth 0.75				Height growth			
	χ^2^	*df*	*p*	%	χ^2^	*df*	*p*	%	χ^2^	*df*	*p*	%
Starting diameter/height	2.0	1	ns		0.2	1	ns		13.8	1	< **0.001**	
Browsing	13.3	1	** < 0.001**	23%	3.6	1	ns	–	0.1	1	ns	–
Insects	0.2	1	ns	–	5.4	1	**0.02**	12%	0.1	1	ns	–
Browsing: insects	1.6	1	ns		1.6	1	ns		0.07	1	ns	

The ‘%’ column shows the percentage difference in growth increment on trees exposed to browsing or insects compared to controls.

**Table 2:** ANOVA (type II) test results for linear mixed model testing the growth response (height and diameter (at both 0.2 m and 0.75 m stem height)) in relation to starting height/diameter and the herbivory treatments (browsing and insects) and their interaction, year 2 (2017) of the experiment. Significant p-values are marked in bold.

Herbivory	Diameter growth 0.2				Diameter growth 0.75				Height growth			
	χ^2^	*df*	*p*	%	χ^2^	*df*	*p*	%	χ^2^	*df*	*p*	%
Starting diameter/height	0.55	1	ns		8.5	1	**0.003**		20.9	1	< **0.001**	
Browsing	27.5	3	** < 0.001**	22%	5.5	3	ns	–	19.5	3	** < 0.001**	9%
Insects	0.1	1	ns	–	5.6	1	**0.02**	4%	2.1	1	ns	–
Browsing: insects	8.7	3	**0.03**		8.1	3	**0.05**		4.6	3	ns	

The ‘%’ column shows the percentage difference in growth increment on trees exposed to browsing or insects compared to controls.

### Insect defoliation

After the first year of the experiment sawfly defoliation resulted in 1.1 mm lower radial growth at 0.75 m (χ^2^ = 5.5, *p* = 0.02), but had no effect on radial growth at 0.2 m stem height nor on height growth (Table 1; Fig. 1), compared to control trees. After the second year, trees exposed to repeated sawfly defoliation (i.e. 2016 and 2017) grew on average 0.7 mm less (corresponding to a 4% reduction in growth) at 0.75 m stem height (χ^2^ = 5.6, *p* = 0.02), compared to trees exposed to sawfly defoliation once (2017) but there was no effect on radial growth at 0.2 m stem height nor on height growth, (Table 2; Fig. 2).

#### **Q2**

Is pine height growth and radial growth affected by how browsing and sawfly defoliation is combined?

There was no effect of combined clipping and sawfly defoliation after the first year (no significant interaction). After the second year, pine radial growth depended on the combination of browsing and sawfly defoliation, i.e. there was a significant interaction between clipping and insect defoliation both at 0.2 m stem height (χ^2^ = 8.7, *p* = 0.03; Table 1; Fig. 1) and at 0.75 m stem height (χ^2^ = 8.1, *p* = 0.05; Table 2; Fig. 2). Repeated sawfly herbivory reduced growth (compared to single defoliation) on un-browsed trees. However, on browsed trees, depending on how sawfly defoliation was combined with browsing, repeated sawfly herbivory resulted in both higher, lower and the same growth as single defoliation. (Table 2; Fig. 2). There was no interaction effect on height growth (Table 2; Fig. 2).

#### **Q3**

Is the combined effect of browsing and sawfly defoliation on pine height and radial growth additive, synergistic or antagonistic?

**Figure 3:**
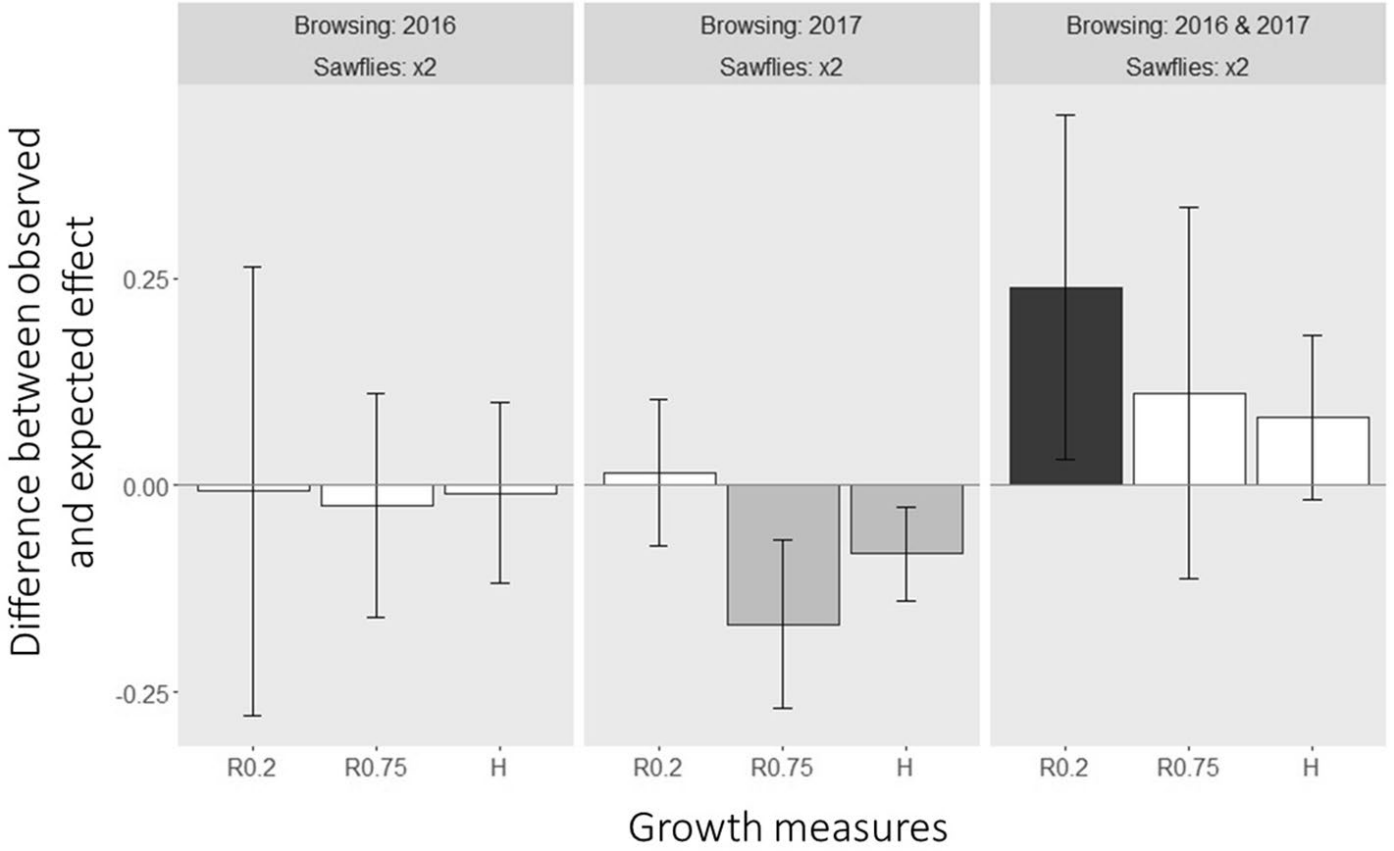
Difference between observed and expected effects of multiple herbivory on tree growth after two growing seasons for trees exposed to clipping and repeated sawfly herbivory (× 2). Expected values are based on the sum of the separate treatments (calculated according to Bansal et al. 2013). Dark grey bars represent synergistic effects, light grey bars antagonistic effects and white bars represent additive effects. Bars and error bars represent the effect size difference (mean and 95% confidence interval around the mean). If the mean and both ends of the confidence interval are above zero growth effects are considered synergistic and if the mean and both ends of the confidence interval is below zero, effects are considered antagonistic. The zero line represents the expected additive effects.

Clipping in 2017 combined with repeated sawfly defoliation (i.e. sawflies in both 2016 and 2017) had antagonistic effects on height growth as well as radial growth at 0.75 m (Fig. 3), i.e. the combined effect of clipping and repeated sawfly defoliation was lower than what could be expected from their separate impacts, and had an additive effect on radial growth at 0.2 m stem height (Fig. 3). Clipping in both 2016 and 2017 combined with repeated sawfly defoliation resulted in synergistic effects on radial growth at 0.2 m, i.e. the combined effect of repeated clipping and repeated sawfly defoliation was higher than what could be expected from their separate impacts, whereas additive effects were found on height growth and radial growth at 0.2 m stem height (Fig. 3). Clipping only in 2016 combined with repeated sawfly defoliation resulted in additive growth effects (Fig. 3).

## Discussion

Our objective was to investigate the single and combined effects of ungulate browsing and insect herbivory on the growth of pine trees. We demonstrate that combined browsing and sawfly defoliation negatively affects pine radial growth but not height growth, and that separately, browsing has a larger impact than sawfly defoliation. Combined browsing and insect herbivory results in both additive and non-additive effects on radial growth of pine trees, depending on the nature of the combination of the two types of herbivory.

We first discuss the two types of herbivory separately to answer our first research question. Starting with browsing, clipping slightly lowered radial growth at 0.2 m after 1 year, but did not affect radial growth at 0.75 m nor height growth (Fig. 1). The lack of a clear effect after 1 year could be because the intensity of browsing was not high enough, or due to a delay in growth response, or both. After the second year, clipping resulted in lower growth at 0.2 m stem height and lower height growth, which corresponds with our expectations and previous studies^5,22^. Turning to sawfly defoliation, we found no overall effects on pine growth, although a small but significant reduction in stem radial growth at 0.75 m after both the first and second growth season (Tables 1, 2; Figs. 1, 2). We assume that a higher intensity of sawfly defoliation is needed to have a substantial overall effect on growth^18^. In addition, trees might be able to compensate for their losses from being exposed to a relatively low amount of sawfly herbivory (only one larval group per tree). Even though we did not observe a strong effect of sawfly defoliation on growth overall, we did observe a difference in radial growth (at both stem heights) between the sawfly treatments after the second year; un-clipped (control) trees exposed to sawflies twice grew less than un-clipped (control) trees exposed to sawflies once, i.e. a negative effect on growth of repeated sawfly defoliation. Such a negative effect has also been previously reported by Ericsson (1980)^19^. This pattern is not consistent among the browsing treatments, as there is an interaction effect (see further in the text, and Fig. 2).

The difference in response of pine trees to browsing (lower stem radial growth at 0.2 m) and insect herbivory (slightly lower radial growth at 0.75 m) is intriguing. The type and spatial pattern of needle removal has previously been demonstrated to cause spatially different growth responses within pine trees^23,24^ and could potentially be the cause of different responses of the pine trees to clipping and sawfly defoliation in this study. At present we cannot explain this difference without a large degree of speculation. To elucidate the mechanisms and explore the consequences for tree growth there is a need for studies especially designed to study these possibly adaptive tree responses.

**Figure 4:**
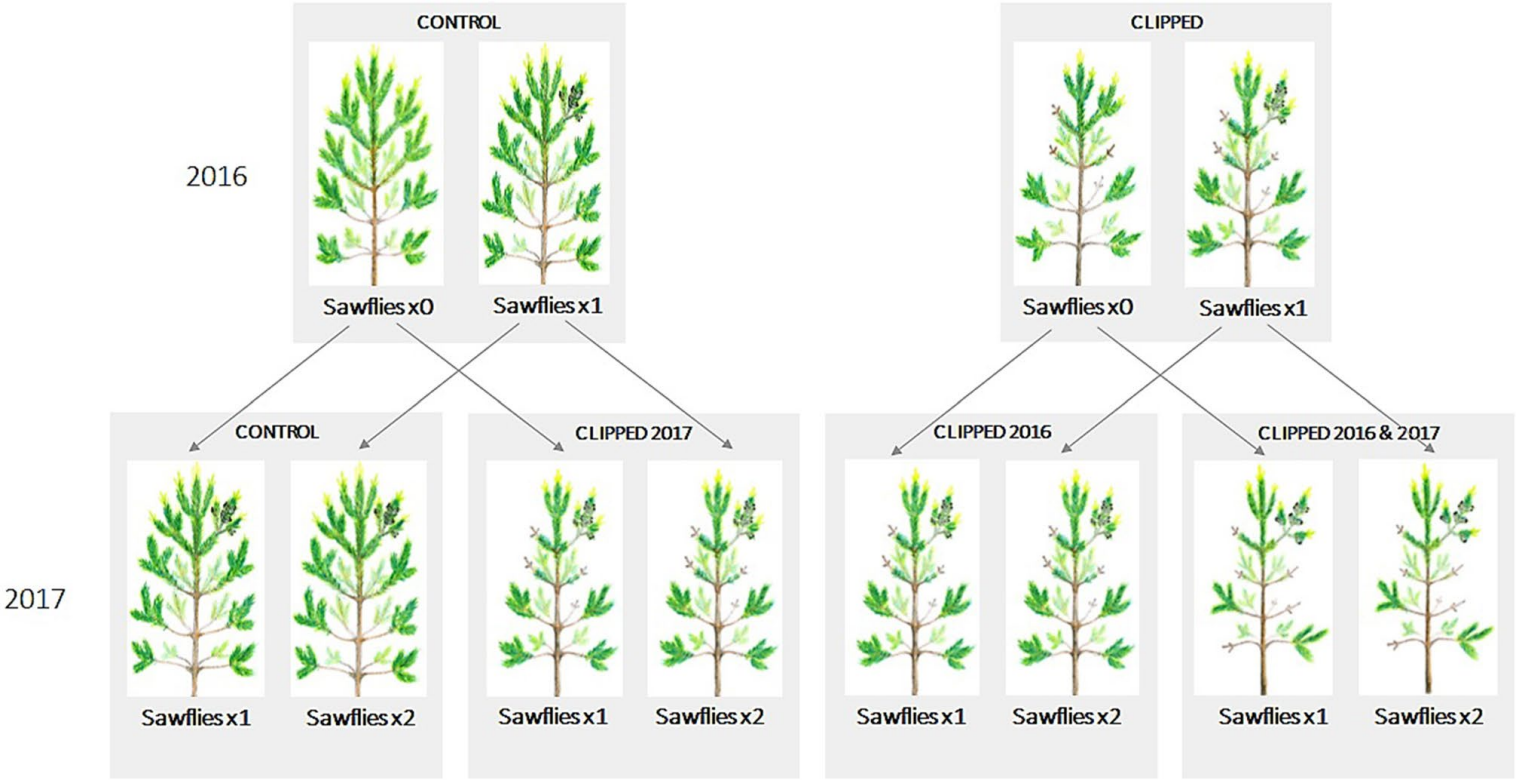
Experimental treatments of pine trees in 2016 and in 2017. Trees were either clipped, exposed to sawflies or both. In 2016 there were four treatments: (i) control (no clipping) and no sawflies, (ii) control (no clipping) and sawflies, (iii) clipped and no sawflies and (iv) clipped and sawflies. In 2017 there were eight treatments: (i) control (no clipping) and sawflies 2017, (ii) control (no clipping) and sawflies 2016 and 2017, (iii) clipped in 2016 and sawflies 2017, (iv) clipped in 2016 and sawflies 2016 and 2017, (v) clipped in 2017 and sawflies 2017, (vi) clipped in 2017 and sawflies 2016 and 2017, (vii) clipped in 2016 and 2017 and sawflies 2017, (viii) clipped in 2016 and 2017 and sawflies 2016 and 2017. Drawings by Fredrika Wrethling.

Concerning the second research question, we found that the combination of browsing and insect defoliation significantly affected radial growth at both 0.2 and 0.75 m stem height, after the second year of the experiment (Table 2; Fig. 2). Radial growth was lower under all ‘combined herbivory’ treatments (compared to control trees) (Fig. 2). Trees seem to be able to partially compensate for the herbivory-caused growth losses (defined by Belsky, 1986^25^ as more growth than zero but less than un-damaged plants) but the magnitude of the compensation depended on the specific combination (i.e. sequence and number of herbivory events) (Fig. 2). Especially trees that were exposed to insects (in 2016) prior to being clipped (in 2017) showed the highest growth (Table 3, Fig. 4). It is possible that sawfly defoliation primed the pines, and thereby initiated higher compensation. Priming is a phenomenon often observed in plants, where one disturbance initiates responses in the plant that able them to respond more/faster to future disturbances^16,17^. Why sawflies would prime the pines while clipping would not, could either be because sawfly defoliation occurs during the active growth period while clipping occurs during dormancy or because of the different patterns of defoliation in browsing and sawfly herbivory. The spatial pattern of defoliation (i.e. which needles and where) has been shown to be of importance for Scots pine growth^23^. Another possible explanation could be inherent differences between natural and artificial herbivory. Studies have shown that plant response can differ between artificial and natural herbivory, due to e.g. chemical cues of herbivore saliva^26,27^, which we did not account for in the clipping treatment. We conclude that the type of herbivory impact pine growth response. Since we only added one sawfly group per tree and still could show a significant, although minor, effect on growth, we argue that single as well as interaction effects on growth could be even stronger in reality as there are commonly more than one group of sawfly larvae per tree.

**Table 3:** Herbivory treatments in 2017.

Treatment	Simulated browsing (clipping)	Insects	Total number of herbivory events	Sequence of herbivory	n_trees_
Control (un-clipped) and insects × 1	No	Once	1	Insect	6
Control (un-clipped) and insects × 2	No	Twice	2	Insect–Insect	7
Clipped × 1 (2016) and insects × 1	Yes–2016	Once	2	Clipping–Insect	8
Clipped × 1 (2016) and insects × 2	Yes–2016	Twice	3	Clipping–Insect–Insect	6
Clipped × 1 (2017) and insects × 1	Yes–2017	Once	2	Clipping–Insect	7
Clipped × 1 (2017) and insects × 2	Yes–2017	Twice	3	Insect–Clipping–Insect	7
Clipped × 2 and insects × 1	Yes–2016 and 2017	Once	3	Clipping–Clipping–Insect	7
Clipped × 2 and insects × 2	Yes–2016 and 2017	Twice	4	Clipping–Insect–Clipping–Insect	8

The first column explains the full treatment, the second which browsing treatment and the third which insect treatment the trees were exposed to. The fourth column contains the number of herbivory events per treatments and the fifth the sequence of herbivory. The sixth column contains the number of trees per treatment (sample size).

We expected mainly additive and antagonistic effects of combined ungulate and sawfly herbivory on pine growth, due to potentially weak indirect effects or priming (Q3)^11,13,17^. Although the prevalence of additive and antagonistic effects was mostly confirmed by our results, we also showed that synergistic effects can occur (Fig. 3) and that the specific combination of herbivory determines whether the response is additive, antagonistic or synergistic. Trees clipped in 2016 and exposed to sawflies twice showed additive responses on all plant traits measured. Trees clipped in 2017 and exposed to sawflies twice showed antagonistic responses for radial growth at 0.75 m stem height and height growth (Fig. 3), again possibly due to sawfly herbivory occurring before clipping, priming the pines to respond to damage prior to browsing. Trees clipped in both years and exposed to sawflies twice showed synergistic or additive effects, with a trend towards synergistic effects on growth, suggesting that when there is a high frequency of herbivory, plants become less able to compensate, impacting growth more.

Our results show interacting effects of insect and ungulate damage on radial growth, and shows that the sequence and number of events of herbivory affect plant responses. We focus on effects of multiple herbivory on tree growth above ground. However, the direct exchange between above and below ground tree biomass might lead to potential effects ignored in our experiment. Including a measure of below-ground biomass or growth could potentially have led to more comprehensive understanding of the divergent responses to different combinations of herbivory that we observed in our experiment. Another important aspect to consider is that Scots pine has a determinate growth habit (growth is partly predetermined). This may influence the outcome of the experiment as there might not be a direct relationship between growth and treatment, and could thus be a factor contributing to the diverging effects in radial growth. However, using a controlled experiment that we designed using knowledge on methods and outcomes of previous studies^5,18,19^ has greatly reduced the risk of not observing comparable tree responses across our treatments. The effects of determinate growth may in fact increase the variation in the data, possibly contributing to the high variation observed in the results. Although, by including starting height and diameter in the models, we account for part of this variation. We did not observe any other major source of herbivory, or any other major disturbance or stress which, in combination with the experimental set up (block design), makes it unlikely that the effect on radial growth is due to other factors. Acknowledging these caveats, we do believe that our study provides valuable insight in the effects of herbivory by taxonomically distant species.

## Conclusions

This study shows that effects on plant growth of multiple herbivores is not straight forward and depends on the sequence and number of damage events. It also corroborates previous studies by showing that the effects of multiple herbivores are not always additive, and confirms our hypothesis that sequential mammal and insect herbivory can cause non-additive effects on tree growth. We conclude that the effect of multiple herbivores on plant growth will depend on the specific herbivores present, how herbivory is combined in time.

## Materials and methods

### Study species

We selected the study system because the species can naturally utilize the same trees, and these interactions commonly occur over prolonged periods of time. Ungulates browse pines in seedling and sapling stage, and the European pine sawfly can occur on pines from sapling to mature stage. Presence of sawfly egg batches has been observed on browsed pines (Klapwijk, personal observations).

#### Scots pine (Pinus sylvestris)

Scots pine is a conifer tree species native to Fennoscandia and one of the most abundant species in Swedish boreal forests. It is also the most widely distributed *Pinus* species in the world, covering large parts of Northern Europe and Asia, as well as being introduced in many areas globally. Scots pine is an economically important tree species, particularly in Fennoscandia.

#### The European pine sawfly (*Neodiprion sertifer*)

The European pine sawfly (*Neodiprion sertifer* Geoffr.; Hymenoptera, Diprionidae) is an herbivorous insect, specialising on pine (*Pinus* spp.). It generally occurs in low population densities but irregularly outbreaks and can cause major damage. Larvae hatch in spring and feed gregariously, commonly in groups of 20–60 individuals^28^. Multiple larval groups per tree is relatively common, especially during outbreaks. Sawflies feed exclusively on previous years’ needles. The European pine sawfly can cause up to 38% reduction in Scots pine volume under severe defoliation^29^.

#### Ungulates

Ungulates can have large effects on forest growth and structure^30,31^ and have been shown to affect growth of Scots pine^5,22,32^. Scots pine is browsed by ungulates mainly in winter^33^. On Scots pine, even low levels of browsing can have significant economic impact^34^.

### Experimental design

The experiments were executed over a period of 2 years (2016, 2017).

### Trees and browsing treatments (Q1)

The experiment was set up in 2016 in Uppsala in Sweden in two young semi-natural forest stands, dominated by pine (Stand 1: 59 52 01.7 N, 18 11 06.4E, Stand 2: 59 58 00.9 N, 18 13 37.0E). 64 un-browsed pines were selected (32 in each stand). The pines were between 7 and 10 years old. We stratified the sampling within each stand to create groups of pine trees growing in a similar microclimate. In each stand, eight groups, each consisting of four pines, were selected, resulting in a total of sixteen groups over the two stands (called ‘blocks’ hereafter). Starting height and diameter at stem height 0.2 m and 0.75 m were measured on all trees before growing season in winter 2016. We measured diameter growth at two stem heights (0.2 m and 0.75 m) to capture potential spatial differences in growth, since disturbances such as herbivory and pruning has been shown to result in divergent growth responses at different stem heights^35^. Diameter was measured with caliper, and marks were made on the stem to ensure that measures were taken at the same place each time. Within each block, trees were randomly assigned a treatment; two of the four selected trees were subjected to clipping (artificial browsing) and the remaining two trees were designated controls (un-clipped). In order to determine the intensity of the applied browsing treatment, we pre-surveyed the browsing damage levels in each stand. The clipping was based on the average level of moose browsing observed (in both stands), which was 50% of the previous season’s lateral shoots (i.e. every second lateral shoot). Trees were clipped while dormant (in early spring 2016) to simulate ungulate winter browsing, the top-shoot was never clipped. Sawfly larvae were added to half of the trees in May 2016 so that the experiment followed a full factorial design (detailed description of sawfly collection and preparation below), yielding four herbivory treatments in total (*i–iv*): *i*) control (no sawflies, no clipping), *ii*) sawflies, *iii*) clipping and *iv*) clipping and sawflies. In seven of the blocks in stand two one additional pine was marked and measured. These seven trees were left un-treated until the end of the experiment as additional full control trees (the term ‘full control’ refers to that the trees were never subjected to clipping or sawfly herbivory during the experiment).

### Trees and browsing treatments (Q2)

In order to further investigate the effect of multiple herbivores on pine growth the second part of the experiment was set up in 2017 in the same stands using the same trees. Trees were subjected to another two browsing treatments to study how different combinations of herbivory (i.e. the sequence and number of herbivory events) affects growth. A second round of clipping simulating winter browsing was applied between the growing seasons. This round of clipping was applied so that half of the previously clipped trees would receive double clipping, and the other trees were left unclipped. Similarly, half of the trees that were previously un-clipped were now clipped and the remaining half of the previously left un-clipped for the second time, which resulted in four overall browsing treatments; 1) control (no clipping), 2) clipped in 2016 only, 3) clipped in 2017 only and 4) clipped consecutively in 2016 and 2017. Sawfly larvae were added to all trees, resulting in a total of eight herbivory treatments (Table 3, Fig. 4). The browsing treatments were applied in such a way that the insect treatments from 2016 were split evenly across the treatments, hence half of the trees within each browsing treatment had been exposed to sawflies both years and the other half only in 2017. Studying the effect of multiple years of browsing is reflecting reality as previously ungulate-damaged pines having a high likelihood of being re-visited and re-browsed^36–38^. After the 2017 growing season all trees were re-measured (both height and stem diameter). At the end of the experiment we cut the stems of a subset of the trees to assess how the external growth measures matched up with patterns in tree ring growth. The method and results of the tree ring analysis are presented in the appendix (Appendix S1) and do not deviate significantly from the results obtained with the external growth measures (Table S1, Fig. S1-S3).

### Collection and preparation of sawfly larvae (Q1 and Q2)

The collection and preparation of sawfly larvae were performed in the same way both years. Sawfly larvae were collected from an area with high pine sawfly density near Oskarshamn in Southern Sweden (57 8 42.4 N, 16 17 55.3E). Larvae were stored in a dark room at 5 ºC, until reaching 2nd instar and thereafter randomly assorted into groups to avoid maternal effects. One larval group was placed on one branch on each tree on the 19th of May 2016 (mean ± standard deviation 49.2 ± 7.3 number of larvae per group) and on the 17th of May in 2017 (mean ± standard deviation 44.2 ± 2.6 number of larvae per group). Larval groups were caged in mesh bags to exclude predation. Larvae were caged with what we deemed to be excessive amount of food, to ensure that the larvae did not run out of food or that it would interfere with their feeding behaviour. The larvae were left to feed until they had spun cocoons (end of June) but were monitored regularly during the whole experiment. Biomass removed by the larval groups was similar across treatments, none of the larval groups did not run out of food, and we observed no major natural herbivory on the experimental trees.

### Clipping rationale

By using clipping, rather than natural ungulate browsing, we can draw more general conclusions on effects of ungulate browsing (both deer and moose). Not using actual browsing (species specific saliva) has the disadvantage of only detecting plant responses to mechanical aspects of the damages, but the advantage of making the results more general towards a variety of browsing mammals. Clipping is a commonly applied method to mimic browsing on trees in general^39,40^ and pine in particular^20,22,32^. Using clipping, rather than natural browsing also allows us to control for both the intensity and timing of damage, which is essential for the experimental set up.

### Statistical analysis

All statistical analyses were performed in R software version 3.6.2^41^. We used linear mixed effect models to analyse the differences in growth, depending on single and combined herbivore treatments. To fit the linear mixed models, we used the lme-function in the nlme package^42^. Herbivory treatments were included as fixed factors and block nested in stand as random grouping factor. The starting value for height and diameter at both stem heights were included as co-variables in the analyses, to take differences between individual trees prior to treatment into account. We used the car package^43^ to calculate the analysis of variance using principles of marginality (Anova, II, car-package). To account for the heterogeneity of variances between browsing treatments we used a variance structure allowing for differences in the variance spread within the treatments (varIdent; car-package^43^). Assumptions of normality and homogeneity were checked by inspecting the residuals and using Levene’s test for homogeneity of variance (leveneTest; car-package^43^), no additional abnormalities were observed. To test if the measured difference in diameter (growth) corresponded to the measured ring width (2016 and 2017) we performed a regression analysis (Fig. S1-S2). Since the additional full control trees were only represented in stand 2, and using only trees from stand 2 in the analysis would considerably reduce the sample size, we did not include the full control trees in the main models. Mean growth increment of the full control trees and visual comparison between growth increment for all treatments can be found in the supplementary material (Appendix S1), and full controls did not appear to differ from un-browsed trees exposed to insects once (Appendix S1, Fig. S4). In order to assess if there was a difference in growth between trees that were full controls (no sawfly herbivory) and trees exposed to single or consecutive sawfly herbivory (but no clipping) we analysed the additional full control trees alongside the un-clipped trees exposed to insects (once or twice), using linear mixed models. Insect treatment (3 levels: full control, sawflies once or sawflies twice) was included as a fixed factor and block nested in stand as random grouping factor. The starting value for height and diameter at both stem heights were included as a co-variable in the analyses. It is important to note that the ‘full control’ trees are only represented within stand 2, whereas the trees exposed to insects are represented in both stands. There was no difference between full control trees and trees exposed once to sawfly herbivory (Supplementary material; Table S2), providing a second motive for excluding the full control trees from the main models.

### Calculating additive, synergistic or antagonistic effect (Q 3)

In order to quantify the effect of multiple herbivores on tree growth we calculated whether the effect of the multiple herbivores on plant growth was additive or non-additive, compared with the single herbivore effects. The calculations were based on the measurements at the end of the experiment (since there was no interaction effect after the first year) for trees exposed to both clipping and repeated sawfly defoliation (Table 3; treatments: ‘clipped × 1 (2016) and insects × 2′, ‘clipped × 1 (2017) and insects × 2′ and ‘clipped × 2 (2016, 2017) and insects × 2′). The observed effect sizes were compared with calculated expected additive effects of herbivory^44,45^. This was done in the following four steps. First, the observed effect of each multiple herbivory treatment (i.e. browsing 2016 + insects × 2, browsing 2017 + insects × 2 and browsing 2016 and 2017 + insects × 2) was calculated by subtracting the mean value ($\overline{x}$ *control*) of the control treatment (i.e. un-browsed trees) from all the individual observed effects, i.e. for each tree (Eq. 1). Second, expected effects for each individual treatment (*Ind*) were calculated by taking the absolute value of the difference between the mean value per treatment ($\overline{x}$ *treatment*) and the mean value of the control ($\overline{x}$ *control*) (Eq. 2), hence representing the single effects of the herbivory treatments. Third, expected additive effects were calculated from the expected individual effects, using the multiplicative risk model (Eq. 3), to avoid over-inflated estimates of the responses. Last, the difference between observed (*Obs*) and expected (*Exp*) multiple effects was calculated (Eq. 4) per tree. Note that the observed effects are one per tree (i.e. the measured values), while the expected effect is one per treatment (calculated according to Eq. 3). The mean values of the difference between the observed and expected effects, along with 95% confidence intervals, were calculated for each treatment. If the mean difference between the observed and expected effects, and both ends of the confidence interval was larger or smaller than zero, effects were considered non-additive.

1 $$Obs = \frac{{Observed\;effect - \overline{x}_{control} }}{{\overline{x}_{control} }}$$

2 $$Ind = \left| { \frac{{\overline{x}_{treatment} - \overline{x}_{control} }}{{\overline{x}_{control} }} } \right|$$

3 $$Exp = Ind_{i} + Ind_{j} - \left( {Ind_{i} \times Ind_{j} } \right)$$

4 $$Obs - Exp$$

## Supplementary Information

Supplementary Information

## Data Availability

The data is available in Dryad Digital repository https://doi.org/10.5061/dryad.bcc2fqz93.
