# Supplementary material for: Interacting effects of insect and ungulate herbivory on Scots pine growth
Source: Sci Rep. 2020 Dec 18;10:22341. doi: 10.1038/s41598-020-79346-3 (PMC7749124; doi:10.1038/s41598-020-79346-3)
Supplement: Supplementary file 1 — Supplementary Information [file 41598_2020_79346_MOESM1_ESM.pdf]

Supplementary material Nordkvist *et al.*

Manuscript title: Interacting effects of insect and ungulate herbivory on Scots pine growth

Michelle Nordkvist[1*], Maartje J. Klapwijk[1], Lars Edenius[2] & Christer Björkman[1]

1 Department of Ecology, Swedish University of Agricultural Sciences, Uppsala, Sweden

2 Department of Wildlife, Fish, and Environmental Studies, Swedish University of Agricultural Sciences, Umeå, Sweden

*Corresponding author: Michelle Nordkvist, michelle.nordkvist@slu.se

Supplementary material – Appendix S1

Tree rings

Tree ring measurements

To be able to more closely examine the observed effects on radial growth we measured tree ring width on a subsample of the trees (n_{trees} = 43). Stems were cut in the field early spring 2018. Thereafter, the stems were marked and cut using a band saw giving disc cross-sections of the upper and lower regions. The disc cross-sections were thereafter planned to provide clear surfaces. Discs were scanned using an HP Scanjet 8200 coupled to WinDENDRO™ (2002) computer software providing numerical information on annual ring width (mm).

Results

The result obtained from the tree ring analysis mostly support the results obtained using the other growth measures. There was a positive relationship between ring width and diameter growth at both 0.2 and 0.75m stem height (Fig. S1 and S2, $p < 0.001$ and $p < 0.001$, respectively). Ring width (sum of the 2016 & 2017 rings) at 0.2m stem was not significantly affected by any of the herbivory treatments, but the χ^2 values pointed in the same direction as the analysis of the caliper measurements (Table S1, Fig, S3). Ring width at 0.75m stem height was affected by the interaction ($\chi^2 = 11.3$, $p < 0.05$), but not clipping nor insects (Table S1, Fig. S3).

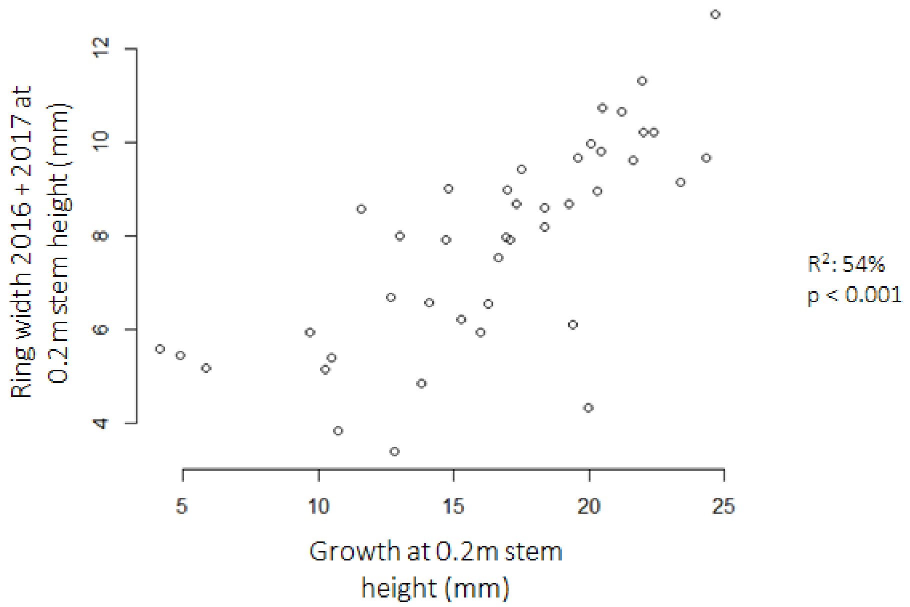

Figure S1. Relationship between radial growth at 0.2m stem height measured with caliper (i.e. difference in diameter over two years) and ring width (the 2016 and 2017 ring widths summed). R-squared (54%) and p-value (< 0.001) displayed in the graph.

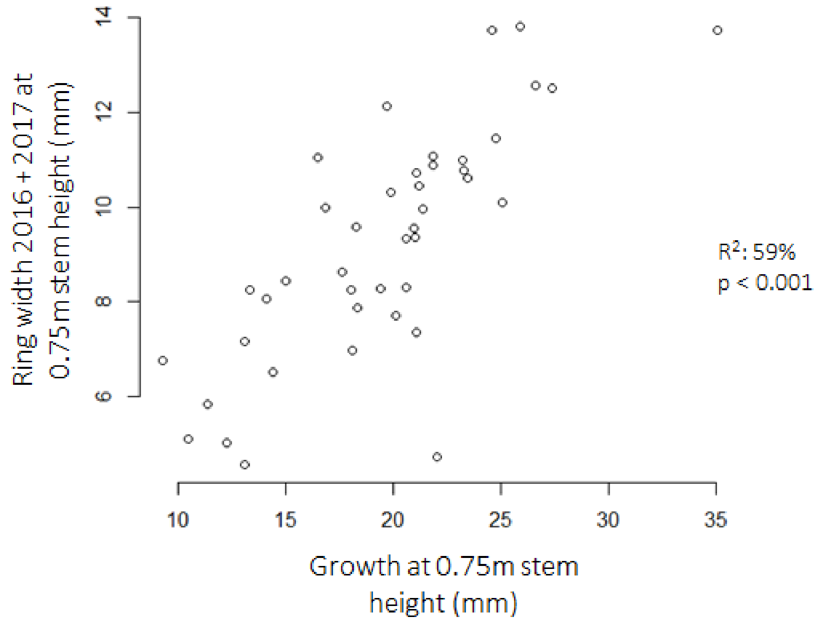

Figure S2. Relationship between radial growth at 0.75m stem height measured with calliper (i.e. difference in diameter over two years) and ring width (the 2016 and 2017 ring widths summed). R-squared (59%) and p-value (< 0.001) displayed in the graph.

Table S1. Anova (type II) test results for linear mixed model testing ring width (2016 + 2017) at 0.2m and 0.75m stem height in relation to starting diameter and the herbivory treatments (browsing and insects) and their interaction.

	Ring width at 0.2m			Ring width at 0.75m		
Herbivory	χ^2	Df	P	χ^2	Df	P
Starting diameter	1.6	1	ns	2.2	1	ns
Browsing	6.3	3	ns	5.1	3	ns
Insects	1.3	1	ns	3.2	1	ns
Browsing : Insects	5.7	3	ns	11.3	3	**0.01**

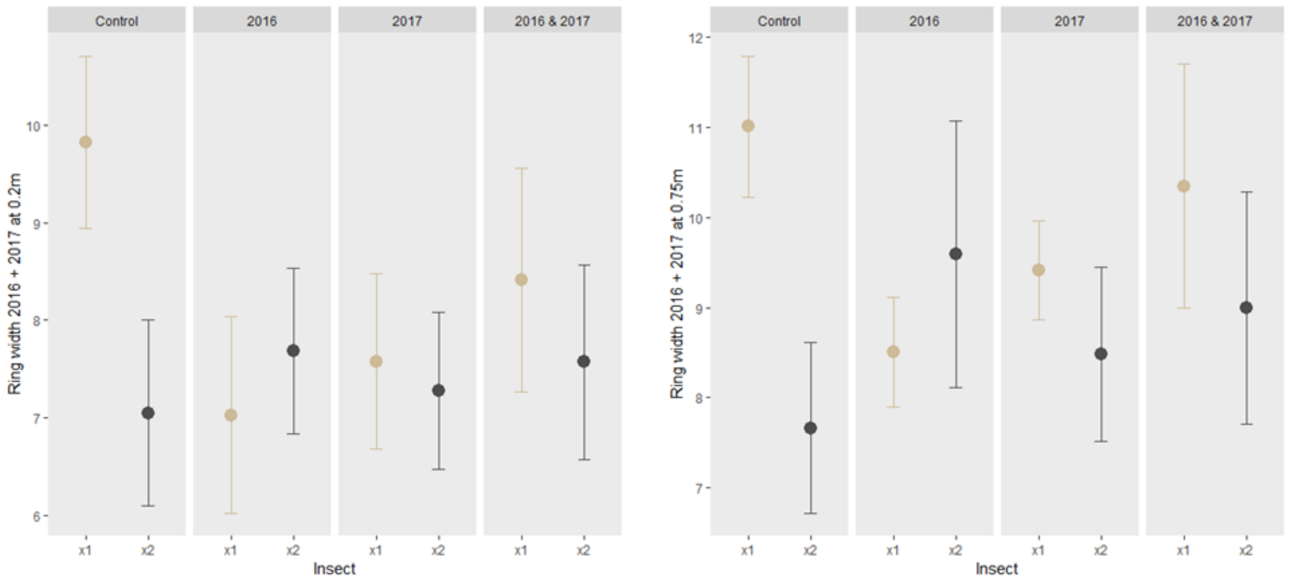

Figure S3. Sum of ring width of rings from 2016 and 2017 at 0.2 and 0.75 m stem height. Mean (mm) of summed ring width (left to right): at 0.2) 9.8, 7.1, 7.0, 7.7, 7.6, 7.3, 8.4, 7.6 and at 0.75) 11.0, 7.7, 8.5, 9.6, 9.4, 8.5, 10.4, 9.0. Panels represent browsing treatments (control, 2016, 2017, 2016 & 2017). X-axis ticks and marker color denotes the insect treatment: Insects once (x1, brown markers and error bars) or insects twice (x2, dark grey markers and error bars). n = 6, 6, 5, 5, 5, 6, 4, 6.

Full control trees

Mean height and diameter growth of the full control trees (± standard deviation) was 111 ± 23 cm, 19.7 ± 3.1 mm (at 0.2 m stem height) and 22.3 ± 5.1 mm (at 0.75 m stem height), respectively.

Table S2. Anova (type II) test results for linear mixed model testing ring height growth and diameter growth at 0.2m and 0.75m stem height in relation to starting diameter and insect treatment (3 levels: full control, insects x1, insects x2) after two years.

	Diameter growth 0.2			Diameter growth 0.75			Height growth		
Insect herbivory	χ^2	*Df*	*P*	χ^2	*Df*	*P*	χ^2	*Df*	*P*
Starting diameter/height	2.5	1	ns	1.7	1	ns	14.7	1	< **0.001**
Insects	1.2	2	ns	4.2	2	ns	1.0	2	ns

Figure S4. Total growth increment of pine trees in 2017 (mean ± SEM) in height (upper panel), in diameter at 0.2 m stem height (middle panel) and in diameter at 0.75 m stem height (lower panel) for all herbivory treatments (left to right: full control (no sawflies, no clipping), un-clipped with sawflies 2017, un-clipped with sawflies 2016 & 2017, clipped in 2016 with sawflies 2016, clipped in 2016 with sawflies 2016 & 2017, clipped in 2017 with sawflies 2016, clipped in 2017 with sawflies 2016 & 2017, clipped in 2016 and 2017 with sawflies 2016 and, clipped in 2016 and 2017 with sawflies 2016 & 2017.

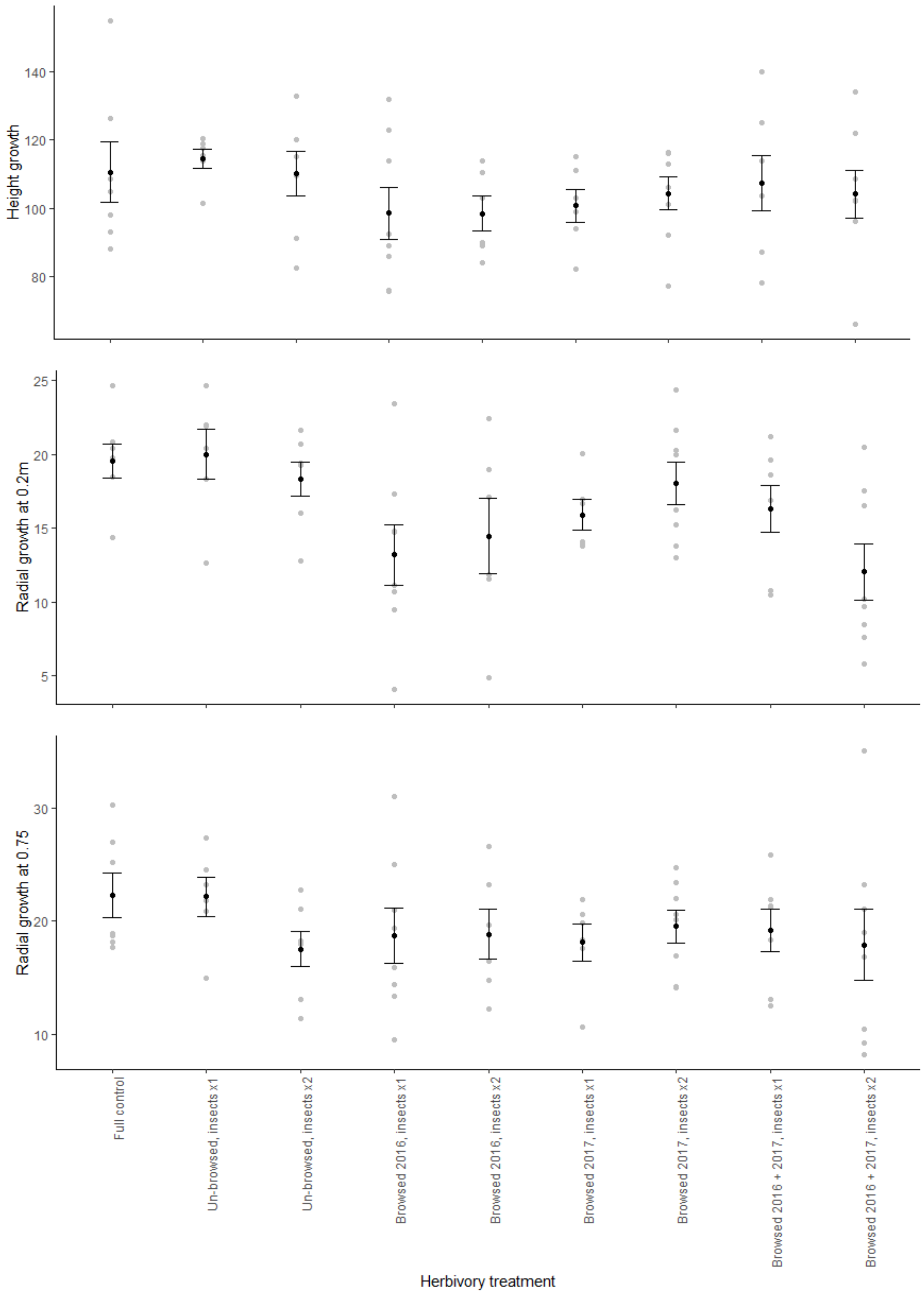

Height growth
140
120
100
80
Radial growth at 0.2m
25
20
15
10
5
Radial growth at 0.75
30
20
10
Full control
Un-browsed, insects x1
Un-browsed, insects x2
Browsed 2016, insects x1
Browsed 2016, insects x2
Browsed 2017, insects x1
Browsed 2017, insects x2
Browsed 2016 + 2017, insects x1
Browsed 2016 + 2017, insects x2
Herbivory treatment
